# Analysis of the accuracy of Z-scores of non-invasive prenatal testing for fetal Trisomies 13, 18, and 21 that employs the ion proton semiconductor sequencing platform

**DOI:** 10.1186/s13039-018-0397-x

**Published:** 2018-08-25

**Authors:** Yuan Tian, Linlin Zhang, Weifang Tian, Jinshuang Gao, Liting Jia, Shihong Cui

**Affiliations:** 1 Department of Clinical Laboratory, The Third Affiliated Hospital of Zhengzhou University, No. 7 Front Kangfu Street, Er’qi District, Zhengzhou, 450052 China; 2 Department of gynaecology and obstetrics, The Third Affiliated Hospital of Zhengzhou University, No. 7 Front Kangfu Street, Er’qi District, Zhengzhou, 450052 China

**Keywords:** Non-invasive prenatal testing (NIPT), False positive, Confined placental mosaicism (CPM), Trisomies

## Abstract

**Background:**

Non-invasive prenatal testing (NIPT) is frequently being used to screen for trisomies 13, 18 and 21 for prenatal diagnosis. However, NIPT performs poorly when compared with invasive testing and thus should not be used to diagnose trisomies. The result of NIPT for an individual woman in most genome-wide methods is calculated as a Z-score. The aim of this study was to assess the correlation between Z-scores of NIPT results and the accuracy of non-invasive prenatal testing.

**Results:**

We evaluated 108 pregnant women with positive NIPT results, which were validated through karyotype analysis of amniotic fluid puncture by means of sequencing, bioinformatics analysis, and follow-up. Utilizing the ion proton semiconductor sequencing platform, we report a performance evaluation of NIPT-positive results at Third Affiliated Hospital of Zhengzhou University of Henan Province, China, by classifying Z-scores as 3 ≤ Z<5, 5 ≤ Z < 9 and Z ≥ 9. The findings indicate that positive NIPT results at Z ≥ 9 have a higher accuracy compared with positive NIPT results at 5 ≤ Z < 9 and 3 ≤ Z<5.

**Conclusions:**

The findings show that Z-scores of NIPT results are closely related to the accuracy of non-invasive prenatal testing. However, false-positive NIPT results at 3 ≤ Z<5 may occur due to confined placental mosaicism (CPM).

## Background

Non-invasive prenatal testing (NIPT) is frequently being applied to screen for trisomies 13, 18 and 21 for prenatal diagnosis. Due to its simple operation and avoidance of amniocentesis, NIPT, as opposed to invasive procedures (chorionic villus sampling (CVS) or amniotic fluid), is regarded as the first choice when screening is required for fetal assessment in pregnant women. Indeed, NIPT is an attractive option because it is procedurally safe for both mother and fetus, can be conducted as early as 10 weeks of gestation, and is highly accurate [1]. Nonetheless, NIPT performs poorly when compared with invasive testing and thus should not be used to diagnose trisomies [2]. Confined placental mosaicism (CPM), which can occur through a mitotic nondisjunction event or through aneuploidy rescue, has one of the greatest obstacles on NIPT result confirmation [3]. CPM is a phenomenon whereby a cytogenetic abnormality, most often trisomy, is confined to the placenta [4]. False-positive NIPT results can occur due to CPM.

Recent NIPT technologies are predominantly based on next-generation sequencing (NGS) [5]. NGS enables rapid and effective clinical testing such as the IONA® test, which uses the Ion proton semiconductor sequencing platform, has a turnaround time, from the start of sample processing to the result, of 3 days [6]. For most genome-wide methods, the result of NIPT for an individual woman is calculated as a Z-score, where the individual sample is compared with a control group of normal (diploid) samples. In some research, an a posteriori risk (PPR) calculator is used to more accurately express the true likelihood of carrying a fetus with a trisomy. For an individual woman, a positive NIPT result with a sensitivity and specificity of more than 99% does not necessarily indicate that she actually has more than a 99% chance of carrying a fetus with a trisomy. The true likelihood depends not only on the NIPT result but also on the prevalence of the anomaly in the population to which she belongs [7], which is expressed as an a priori risk. Thus, to properly counsel women about a positive result by cell-free fetal DNA screening, it can be useful to express the result as a personalized PPR, which takes the woman’s a priori risk into account. Research shows that PPR is effectively independent under all conditions for Z-scores above 6, and high PPR for low a priori risk can only be reached at Z-scores> 5 [8].

In the present study, we report a performance evaluation of NIPT-positive results in our hospital by classifying Z-scores as 3 ≤ Z<5, 5 ≤ Z < 9 and Z ≥ 9. The results of our study indicate that positive NIPT results with Z-scores of Z ≥ 9 have higher accuracy compared with positive NIPT results with Z-scores of 3 ≤ Z<5 and 5 ≤ Z < 9.

## Methods

### Patients

Written informed consent was obtained from each patient. The study was a retrospective analysis of NIPT-positive results in singleton pregnancies from January 2015 to December 2017 at Third Affiliated Hospital of Zhengzhou University of Henan Province, China. Positive NIPT results would be validated through postpartum follow-up of patients, involving the results of karyotype analysis via amniotic fluid puncture, including trisomies 13, 18, and 21 (T21, T18 and T13); 108 cases were validated in this manner.

### Sequencing process

Maternal peripheral blood (5 ml) was collected into an ethylenediaminetetraacetic acid (EDTA) tube at 12 + 0 to 25 + 6 weeks of gestation. The blood samples were stored at 4 °C immediately after collection. Plasma was isolated within 8 h through a two-step centrifugation protocol, after which the samples were stored at − 80 °C. Cell-free DNA extraction, library construction, sequencing, and bioinformatics analysis were performed according to a previous study [6].

### Bioinformatics analysis

Sequencing data were analyzed by using the ion proton semiconductor sequencing platform and software from Capitalbio Corporation. The binary hypothesis Z-score of particular chromosomes in each sample was determined, as reported previously [7]. To assess fetal risk of T21, T18 and T13, sample with a Z-score ≥ 3 for these chromosomes was classified as positive. We classified the positive samples into three groups according to Z-score = 5 and Z = 9 among them. Group 1 contained samples with NIPT results of 3 ≤ Z<5. Group 2 contained samples with NIPT results of 5 ≤ Z<9. Group 3 contained samples with NIPT results of Z ≥ 9.

### Karyotype analysis and amniotic fluid puncture

For those with positive NIPT results, karyotype analysis and amniotic fluid puncture were recommended for validation. Amniotic fluid puncture was performed as routinely described; karyotype analysis was performed according to the International System for Human Cytogenetic Nomenclature guidelines [9].

### Follow-up

Follow-up for NIPT-positive cases was performed via telephone. The follow-up duration was 56 days after delivery.

## Results

### Patient characteristics

A total of 21,114 pregnant women were tested by NIPT from 2015 to 2017, with 202 having positive results. According to follow-up, cases of induced labor that was not validated through other invasive methods were removed. Overall, 108 NIPT-positive women who were validated via karyotype analysis and amniotic fluid puncture, with a mean maternal age of 33.5 (19–48 years), were included in this study. Gestational age was from 12 weeks to 25 + 6 weeks at blood sample collection. Because the fetal DNA fraction in NIPT needs to be higher than 4% for a singleton and 8% for twins, pregnant women tend to accept NIPT at more than 16 weeks to obtain a higher fetal fraction. As cases of NIPT-positive results should be validated through karyotype analysis and amniotic fluid puncture, we would suggest that the patients accept NIPT within 20 weeks. The patient characteristics are listed in Table 1.

**Table 1:** Basic Characteristics of 108 NIPT-Positive Patients

Characteristics	Numbers
Maternal age(years)	
≤ 20	1
20–24	5
25–29	31
30–34	21
35–39	35
40–45	13
≥ 45	2
Mean	33.5
Range	19–48
Gestation at NIPT tests(weeks)	
12–12 + 6	4
13–13 + 6	9
14–14 + 6	5
15–15 + 6	8
16–16 + 6	17
17–17 + 6	14
18–18 + 6	14
19–19 + 6	17
20–20 + 6	5
21–21 + 6	6
22–22 + 6	2
23–23 + 6	5
24–24 + 6	0
25–25 + 6	2

## Relationship of NIPT-positive results group 1 with karyotype analysis and amniotic fluid puncture

In total, 18 NIPT-positive cases were included in Group 1 (3 ≤ Z<5); 15 cases with false-positive results were validated through karyotype analysis and amniotic fluid puncture. Conversely, 3 true-positive cases were validated through karyotype analysis and amniotic fluid puncture, with a positive predictive value (PPV) of only 16.67%. The NIPT-positive cases in Group 1 are listed in Table 2. By analyzing the data of the 3 true-positive NIPT cases, we found that the fetal DNA fraction estimation for samples YC151439 and YC173711 was less than 4%; the fetal DNA fraction estimation for sample YC174141 was 6.4%, which was less than 8%.

**Table 2:** The 18 NIPT-Positive Cases in Group 1 (3 ≤ Z<5)

No.	Sample ID	Z-scores	NIPT results	Fetal karyotype
1	YC150550	3.330	T21	46,XN
2	YC150816	4.342	T13	46,XN
3	YC151439	3.643	T21	47,XN + 21
4	YC161217	4.056	T13	46,XN
5	YC163341	4.778	T13	46,XN
6	YC164267	3.968	T13	46,XN
7	YC165157	3.392	T21	46,XN
8	YC165602	3.362	T18	46,XN
9	YC167031	3.075	T21	46,XN
10	YC171503	3.207	T21	46,XN
11	YC173711	4.951	T21	47,XN + 21
12	YC174141	3.242	T21	47,XN + 21
13	YC175988	3.713	T21	46,XN
14	YC175996	4.111	T18	46,XN
15	YC176477	3.351	T18	46,XN
16	YC176775	3.620	T21	46,XN
17	YC177355	3.263	T21	46,XN
18	YC177383	3.697	T13	46,XN

## Relationship of NIPT-positive results for group 2 with karyotype analysis and amniotic fluid puncture

A total of 34 NIPT-positive cases were in Group 2 (5 ≤ Z<9). Four cases of false positivity were validated through karyotype analysis and amniotic fluid puncture. In contrast, 30 true-positive results were validated via karyotype analysis and amniotic fluid puncture, with a PPV of 88.24%. Table 3 lists the positive NIPT cases in Group 2.

**Table 3:** The 34 NIPT-Positive Cases in Group 2 (5 ≤ Z<9)

No.	Sample ID	Z-scores	NIPT results	Fetal karyotype
1	YC150539	5.570	T21	47,XN + 21
2	YC150431	5.960	T18	47,XN + 18
3	YC150725	7.463	T21	47,XN + 21
4	YC152284	8.249	T21	47,XN + 21
5	YC153184	8.532	T21	47,XN + 21
6	YC160356	8.830	T21	46,XN
7	YC161880	8.188	T21	47,XN + 21
8	YC163276	5.467	T18	47,XN + 18
9	YC163808	6.104	T21	47,XN + 21
10	YC164065	5.219	T18	47,XN + 18
11	YC164811	8.489	T21	47,XN + 21
12	YC165074	6.677	T21	47,XN + 21
13	YC165729	5.944	T21	47,XN + 21
14	YC166180	6.150	T21	47,XN + 21
15	YC166202	6.220	T18	47,XN + 18
16	YC166253	5.931	T21	46XX + 21,der(14;15)(q10,q10)
17	YC166422	8.945	T13	46,XN
18	YC167160	5.710	T18	46,XX/47,XX + 18(28:2)
19	YC167331	7.790	T21	47,XN + 21
20	YC168025	6.840	T21	47,XN + 21
21	YC170096	8.280	T21	47,XN + 21
22	YC171234	6.119	T21	47,XN + 21
23	YC171535	7.273	T21	47,XN + 21
24	YC171540	6.019	T21	47,XN + 21
25	YC173331	5.330	T21	47,XN + 21
26	YC173539	7.514	T21	47,XN + 21
27	YC173759	6.834	T21	47,XN + 21
28	YC175051	6.908	T21	47,XN + 21
29	YC175492	8.646	T18	47,XN + 18
30	YC175694	6.704	T13	47,XN + 13
31	YC176740	7.803	T21	47,XN + 21
32	YC177345	5.178	T21	46,XN
33	YC176881	5.566	T21	47,XN + 21
34	YC177644	8.523	T21	47,XN + 21

**Fig. 1:**
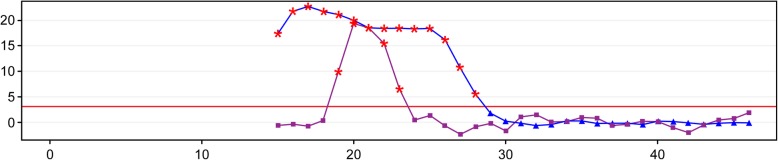
The Distribution Curve of Z-scores for Chromosome 21 of Sample YC160356

**Fig. 2:**
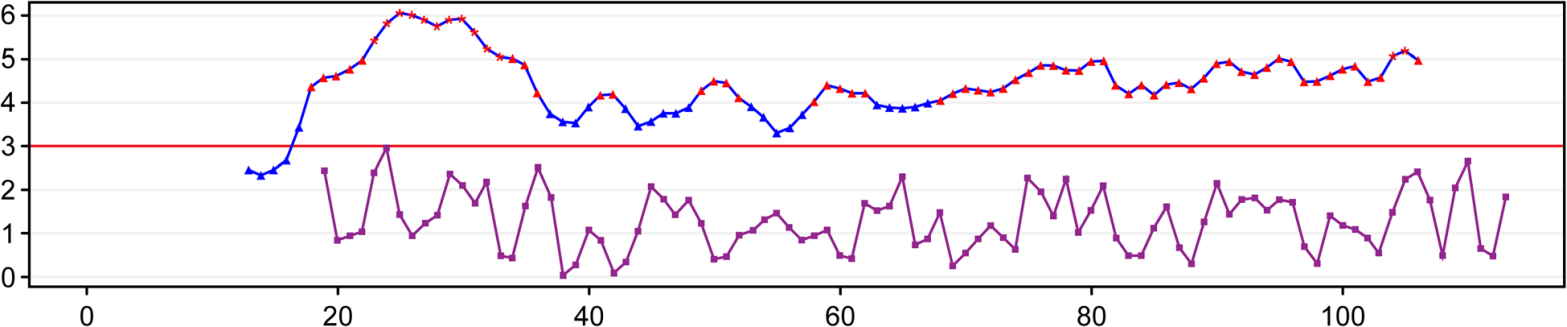
The Distribution Curve of Z-scores for Chromosome 13 of Sample YC166422

**Fig. 3:**
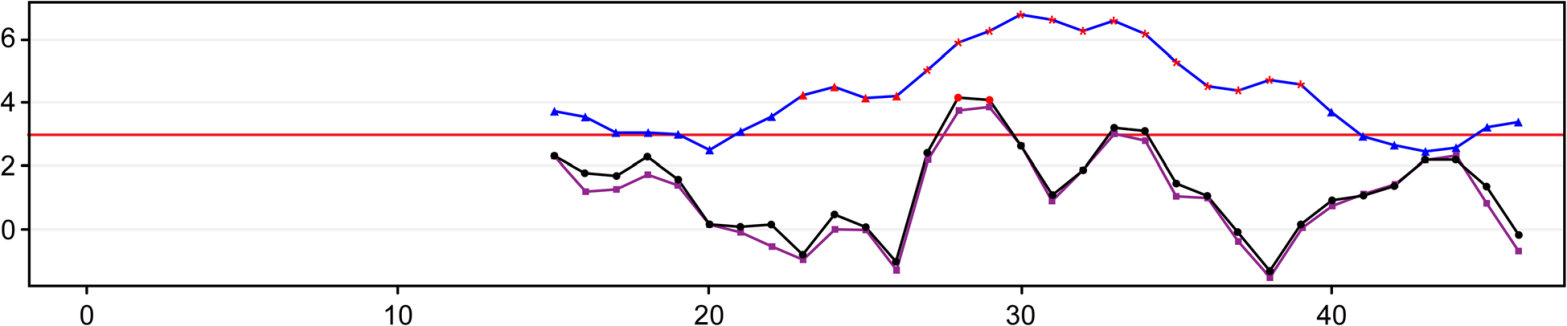
The Distribution Curve of Z-scores for Chromosome 21 of Sample YC177345

Based on analysis of the data for the 4 false-positive cases, sample YC167160 was validated as CPM through karyotype analysis and amniotic fluid puncture (Table 3). Sample YC160356 was possibly affected by chromosome duplication from the maternal fragment (Fig. 1). The false-positive NIPT result for samples YC166422 and YC177345 might have resulted from a small region of chromosome duplication (Fig. 2 and Fig. 3).

## Relationship of NIPT-positive results for group 3 with karyotype analysis and amniotic fluid puncture

Group 3 (Z ≥ 9) comprised 56 cases of NIPT positivity. Only one false-positive case was validated by karyotype analysis and amniotic fluid puncture. For sample YC175377, a unique sample with a false-positive result, a unique sample with a false-positive result, the discordant NIPT result was caused by a vanished twin, as revealed by NIPT in the 4 weeks during follow-up. In total, 55 true-positive cases were validated through karyotype analysis and amniotic fluid puncture; the PPV was 98.12%. The positive NIPT cases in Group 3 are listed in Table 4.

**Table 4:** The 56 NIPT-Positive Cases in Group 2 (Z ≥ 9)

No.	Sample ID	Z-scores	NIPT results	Fetal karyotype
1	YC151198	11.203	T21	47,XN + 21
2	YC151297	11.605	T21	47,XN + 21
3	YC151374	9.306	T21	47,XN + 21
4	YC151424	19.541	T21	47,XN + 21
5	YC151577	14.882	T21	47,XN + 21
6	YC151725	12.367	T21	47,XN + 21
7	YC152585	18.082	T21	47,XN + 21
8	YC153464	10.126	T21	47,XN + 21
9	YC153533	11.864	T21	47,XN + 21
10	YC160344	14.298	T21	47,XN + 21
11	YC161058	10.8	T21	47,XN + 21
12	YC161066	21.457	T21	47,XN + 21
13	YC161368	13.275	T21	47,XN + 21
14	YC162688	9.707	T21	47,XN + 21
15	YC162822	16.21	T18	47,XN + 18
16	YC162943	11.347	T21	47,XN + 21
17	YC163223	13.694	T21	47,XN + 21
18	YC163261	13.627	T21	47,XN + 21
19	YC163310	13.977	T18	47,XN + 18
20	YC163400	11.603	T13	47,XN + 13
21	YC164204	10.546	T21	47,XN + 21
22	YC164355	11.135	T21	47,XN + 21
23	YC164722	13.955	T21	47,XN + 21
24	YC164999	13.661	T21	47,XN + 21
25	YC165003	11.899	T18	47,XN + 18
26	YC165723	10.192	T21	47,XN + 21
27	YC165823	10.346	T18	47,XN + 18
28	YC165859	11.962	T18	47,XN + 18
29	YC166626	9.374	T13	47,XN + 13
30	YC167544	11.253	T18	47,XN + 18
31	YC168149	11.004	T21	47,XN + 21
32	YC170133	12.085	T21	47,XN + 21
33	YC170411	12.536	T21	47,XN + 21
34	YC170724	10.017	T21	47,XN + 21
35	YC170829	12.456	T21	47,XN + 21
36	YC170974	11.555	T21	47,XN + 21
37	YC171134	9.885	T21	47,XN + 21
38	YC171423	11.41	T21	47,XN + 21
39	YC172015	18.693	T21	47,XN + 21
40	YC172946	20.224	T21	47,XN + 21
41	YC173326	9.674	T21	47,XN + 21
42	YC173370	17.431	T21	47,XN + 21
43	YC173573	26.587	T21	47,XN + 21
44	YC173742	9.054	T21	47,XN + 21
45	YC174011	13.767	T21	47,XN + 21
46	YC174110	11.488	T21	47,XN + 21
47	YC174589	16.765	T21	47,XN + 21
48	YC175377	12.367	T18	46,XN
49	YC175406	11.424	T21	47,XN + 21
50	YC175583	9.772	T21	47,XN + 21
51	YC175824	13.609	T21	47,XN + 21
52	YC175903	12.095	T21	47,XN + 21
53	YC176469	11.443	T18	47,XN + 18
54	YC177386	25.448	T18	47,XN + 18
55	YC176915	12.225	T21	47,XN + 21
56	YC178093	18.509	T18	47,XN + 18

Among all of the 21,114 patients accepting NIPT, two cases of false-negative NIPT results were found. However, those with false-negative NIPT results refused to accept further validation of aborted tissue.

## Discussion

NIPT has been widely used in the last few years for prenatal screening of T21, T18 and T13. Although PPR calculation suggests that high PPR for low a priori risk can only be reached at Z-scores> 5 [7], large-scale clinical studies to validate the hypothesis are lacking. The present study including 108 NIPT-positive cases was performed to investigate the relationship between NIPT Z-scores and accuracy of trisomy 13, 18, and 21 diagnosis.

The PPV is much more meaningful to clinicians and patients than the commonly reported measures of sensitivity and specificity [10]. The PPV referred to here is a population-based figure that reports the chance prior to testing that an abnormal test result is actually reflective of the karyotype of the fetus. Except for the PPV, neither sensitivity nor specificity reflects the prevalence of a disorder in the population [10]. Based on the results of the present study, a sample with Z ≥ 9 has higher accuracy than does a sample with 3 ≤ Z<5 or 5 ≤ Z<9. Although the PPV rate in Group 2 (88.24%) was apparently higher that of Group 1 (16.67%), this approach cannot be used for diagnosis because of the relatively high false-positive rate (11.76%). Nonetheless, positive results at Z ≥ 9 are significant for diagnosing trisomies 13, 18 and 21; However, karyotype analysis is necessary for validating trisomies due to the exist of sample YC175377, whose discordant NIPT result may be caused by a vanished twin. There are many biological reasons for discordant results that can be of either fetal or maternal origin. Contributing fetal factors include an insufficient or absent fetal fraction, confined placental mosaicism and the presence of a vanishing twin [11]. For example, Hall AL et al. reported a case that confirmed positive cell-free fetal DNA (cffDNA) testing for trisomy 13, revealing confined placental mosaicism [12]. In addition, Chen C et al. reported a pregnancy with discordant fetal and placental chromosome 18 aneuploidies, as revealed by invasive and noninvasive prenatal diagnoses [13].

CPM is one of the major factors causing false positivity. cffDNA originates from chorionic villi in the placenta, where the constant turnover of cytotrophoblasts induces apoptosis and cffDNA release into the maternal blood. The cytotrophoblast layer of chorionic villi is not always representative of the fetus, as it is derived from the trophoblast of the blastocyst; in contrast, the fetus is derived from the inner cell mass (epiblast) [14]. Postzygotic mitotic division errors in chromosomally normal and abnormal conceptuses will lead to chromosomal mosaicism [15]. Most often, mosaicism is characterized as CPM, in which the abnormal cell line is confined to the cytotrophoblast and/or the mesenchymal core of the chorion villi, whereas the fetus has a normal karyotype [15]. If abnormal cells are restricted to the cytotrophoblast layer and are not present in the mesenchymal core or fetus, such a case is categorized CPM and depending on the extent of mosaicism, will possibly cause a false-positive NIPT result. According to the above, we suggest that the occurrence of CPM may slightly elevate a Z-score for NIPT, higher than 3 but lower than 5.

In addition, estimation of the fetal DNA fraction is important for the accuracy of NIPT results. The definition of the fetal fraction of circulating cell-free DNA in maternal blood is the amount of cffDNA divided by the amount of total cell-free DNA. The circulating cell-free DNA (cfDNA) in a pregnant woman is a mixture of predominant maternal DNA derived from her hematopoietic system and fetal DNA released through apoptosis of cytotrophoblast cells during fetal development [16]. A fetal DNA concentration of less than 4% in a maternal plasma sample would suggest a potential issue present in the quality control (QC) step [17]. Methods for calculating the fetal fraction differ between laboratories. We are aware of six different methods based on methylation [18], single-nucleotide polymorphisms [19], quantitative polymerase chain reaction of the Y-chromosome (only for male fetuses) [20], autosomal regional read counts calculated by SeqFF [21], differences in length between cffDNA and maternal cffDNA [21] and single reads nucleosome-based fetal fraction [22]. The fetal fraction is also related to maternal weight and body mass index. For example, the increased turnover of adipocytes in obese women increases the amount of maternal cell-free DNA and decreases the cffDNA fraction [23]. Furthermore, gestational age influences the fetal fraction. Some research has found that levels of cffDNA increase at 0.1% per week between 10 and 21 weeks of gestation and at 1% per week beyond 21 weeks of gestation [24]. Because the limited amount of fetal DNA molecules to be detected and analyzed may give rise to a false-negative result, we deduce that the Z-scores of samples YC151439, YC173711 and YC174141 were less than 5 because of the estimation of a low fetal DNA fraction.

Regardless, maternally derived chromosomal abnormalities (likely also submicroscopic abnormalities) constitute a rare case of maternal occult malignancy and usually cause false-positive results [25]. Our study validated the hypothesis regarding the false-positive NIPT result for sample YC160356 (Fig. 1).

## Conclusions

NIPT is feasible for prenatal screening of T13, T18 and T21, with higher sensitivity and specificity compared with conventional methods. However, Z-scores of NIPT results are closely related to the accuracy of non-invasive prenatal testing that employs the ion proton semiconductor sequencing platform. NIPT results with low Z-scores should not be used for diagnosis, which should be performed using invasive testing. However, the relationship between Z-scores of NIPT and CPM lacks validation by clinical trials due to the influence of chorionic villi in the placenta.

## Abbreviations

cfDNA: Circulating cell-free DNA
CPM: Confined placental mosaicism
EDTA: Ethylenediaminetetraacetic acid
NIPT: Non-invasive prenatal testing
QC: Quality control
